# A call for better care: the impact of postnatal contact services on women’s parenting confidence and experiences of postpartum care in Queensland, Australia

**DOI:** 10.1186/s12913-014-0635-9

**Published:** 2014-12-20

**Authors:** Yvette D Miller, Aimée C Dane, Rachel Thompson

**Affiliations:** School of Public Health and Social Work, Queensland University of Technology, Brisbane, QLD 4059 Australia; School of Psychology, The University of Queensland, Brisbane, QLD 4072 Australia; The Dartmouth Institute for Health, Policy and Clinical Practice, Dartmouth College, Hanover, NH 03755 USA

**Keywords:** Postnatal care, Postpartum care, Maternal-child nursing, Program evaluation, Maternal confidence, Maternal satisfaction

## Abstract

**Background:**

Universal postnatal contact services are provided in several Australian states, but their impact on women’s postnatal care experience has not been evaluated. Furthermore, there is lack of evidence or consensus about the optimal type and amount of postpartum care after hospital discharge for maternal outcomes. This study aimed to assess the impact of providing Universal Postnatal Contact Service (UPNCS) funding to public birthing facilities in Queensland, Australia on women’s postnatal care experiences, and associations between amount and type (telephone or home visits) of contact on parenting confidence, and perceived sufficiency and quality of postnatal care.

**Methods:**

Data collected via retrospective survey of postnatal women (N = 3,724) were used to compare women who birthed in UPNCS-funded and non-UPNCS-funded facilities on parenting confidence, sufficiency of postnatal care, and perceived quality of postnatal care. Associations between receiving telephone and home visits and the same outcomes, regardless of UPNCS funding, were also assessed.

**Results:**

Women who birthed in an UPNCS-funded facility were more likely to receive postnatal contact, but UPNCS funding was not associated with parenting confidence, or perceived sufficiency or perceived quality of care. Telephone contact was not associated with parenting confidence but had a positive dose–response association with perceived sufficiency and quality. Home visits were negatively associated with parenting confidence when 3 or more were received, had a positive dose–response association with perceived sufficiency and were positively associated with perceived quality when at least 6 were received.

**Conclusions:**

Funding for UPNCS is unlikely to improve population levels of maternal parenting confidence, perceived sufficiency or quality of postpartum care. Where only minimal contact can be provided, telephone may be more effective than home visits for improving women’s perceived sufficiency and quality of care. Additional service initiatives may be needed to improve women’s parenting confidence.

## Background

Some Australian women, especially first-time mothers, lack parenting confidence in the early postnatal period [1]. Parenting confidence is associated with quality of child care [2,3], positive mother-infant interactions, the ability to recognise and respond to infant signals [2,4,5], positive parenting, and parental involvement [2]. Increasing maternal confidence is a target in efforts to foster positive parenting behaviours and potentially reduce risk to the child [2], and has been shown to influence positive parenting behaviours for children in the first year of life regardless of other factors such as maternal depression and social support [6]. Women perceive access to health professional support as important for improving confidence to care for their babies [1] but remain dissatisfied with postnatal care in Australia and report unmet needs and confusion about where to get help in the postpartum period [7-11]. One response has been to design and implement policies for provision of universal postnatal contact following hospital discharge.

There is a lack of consensus on the optimal amount and mode of postpartum health care after hospital discharge and systems for providing community based postpartum care after hospital discharge vary widely across the world and within Australia [12]. However, health care policies and guidelines in a number of Australian states provide specific recommendations for universal postnatal contact. Universal home visiting within 10 days of birth is recommended in Western Australia [13], and within two weeks of birth in New South Wales, South Australia and Victoria [13-16]. In Queensland, one health professional contact by either telephone or home visit within 10 days of hospital discharge was recommended for all women who birth in public facilities from 2008 [17]. Publicly-funded community based post-birth care may be provided by domiciliary midwives employed by birthing facilities, and/or by child and family health nursing services routinely available to new mothers and their children [18]. Generally, the purpose of these visits is to assess maternal and infant risk and provide brief interventions (e.g., breastfeeding support) and/or refer to specialist services as needed.

There is currently no evidence supporting a population-level impact of government funding for implementing universal postnatal care services on the amount or type of postnatal care received by women, or on their parenting confidence and evaluations of care. One study evaluating the impact of providing a universal postnatal program found that neither a postnatal telephone call nor a postnatal home visit from a public health worker was associated with continuation of breastfeeding to 4 weeks [19]. Another found that universal provision of postnatal nurse home visiting decreased use of emergency hospital care for infants in the first year of life [20]. The impact of implementing universal postnatal care services on other aspects of women’s postnatal care and well-being has not been evaluated.

Evidence from randomised controlled trials examining the effects of specific types of postnatal support for low-risk women suggests that benefits of universal provision of postnatal care for maternal parenting confidence are unlikely [21]. Shaw et al. found no impact of telephone calls or home visits by a public health nurse or midwife within 1–2 weeks of discharge on primiparous women’s knowledge, attitudes or skills [21]. Compared to a telephone call, home visits by a public health nurse within 10 days of discharge have been found not to affect maternal confidence [22].

There is conflicting evidence of the effect of home visits on women’s satisfaction with care, and no evidence from Australian samples [23]. For low-risk U.S. women, a home visit within 48–72 hours of discharge resulted in higher maternal satisfaction with care (including satisfaction with amount of time spent, convenience, quality of advice, caring attitude of provider and overall care) than out-of-home clinic visits [24,25] although findings from a study of Canadian low-risk women found no difference in satisfaction when comparing home and clinic visits at 3–4 days post-discharge [26]. In women discharged from hospital early in Syria, there was no difference in overall satisfaction with the postnatal experience between women randomised to receive no home visits, one, or four home visits in the first month after discharge, although satisfaction with postnatal care provision was not specifically assessed [27].

The relative effects of home visits and telephone calls on women’s satisfaction with postnatal care have not been directly assessed, although Goulet, D’Armour and Pineault reported higher rates of perceived *usefulness* of contact among women receiving home visits than those who received a telephone call both within and beyond 72 hours of hospital discharge [28]. Dose–response associations between telephone or home visit postnatal contact and parenting confidence and perceived quality of care remain largely unknown. Population-level evidence about the most effective format (telephone vs. home visiting) and amount of contact for improving women’s parenting confidence and satisfaction with care is needed to guide policy decisions about continued investment in universal postnatal care provision or alternative systems for postpartum care. As highlighted by the WHO expert panel on postpartum and postnatal care, there is need for evidence on the effects of existing models of postpartum care delivery, and of different models (timing and number of postnatal visits) in relation to women’s expectations and experiences with care in the postnatal period [29].

This paper describes the findings of an independent evaluation of the impact of funding a Universal Postnatal Contact Service (UPNCS) across public health services in Queensland, Australia. Maternal self-reported data were used to evaluate the impact of UPNCS funding on postnatal contact, parenting confidence, perceived sufficiency and perceived quality of postnatal care, and to determine the factors (including type and amount of postnatal contact) associated with those same outcomes.

## Methods

### Participants

Participants were respondents to the *Having a Baby in Queensland Survey 2010*, which retrospectively assessed consumers’ experiences of care during pregnancy, labour and birth, and after birth. The sampling frame was databases of compulsory birth notification and registration records, held by the *Queensland Registry of Births, Deaths and Marriages*. All women who had a live birth in Queensland, Australia between 1st February and 31st May 2010, and were not found to have had a baby that died since birth, were eligible.

The entire eligible population was sent a survey package by the *Queensland Registry of Births, Deaths and Marriages* four to five months after birth (in four sequential mailings). The package included an information sheet, a paper survey, and participation instructions in 19 non-English languages. Women could (i) complete and return the paper survey using a reply-paid envelope, (ii) complete the same survey online, or (iii) complete only core survey items via telephone (free call) with a female interviewer and, if necessary, a translator from the Australian Government *Translating and Interpreting Service*. All women were gifted a pen and those who completed the survey within a specified timeframe were invited to enter a draw to win one of four $200 gift cards. All women were sent a reminder to complete the survey two weeks after the initial mailing. To protect women’s privacy, identifying details of women in the sample were never released outside of the *Queensland Registry of Births, Deaths and Marriages*, precluding any tailored follow-up of non-respondents or direct analysis of respondent bias. Further details about the procedure can be found elsewhere [30,31]. The sample for the current study comprised survey respondents that had birthed in a public facility (hospital or co-located birth centre) and had complete data on key variables of interest.

### Intervention

In 2008, the Queensland Government began staged funding for a Universal Postnatal Contact Service (UPNCS) to public birth facilities in Queensland, Australia. The initiative had three components: (1) universal antenatal screening for depression, domestic violence, drug and alcohol use, psycho-social wellbeing and tobacco use, (2) establishment of ‘Newborn and Family Drop-in Centres’ across the state, and (3) universal provision of at least one postnatal contact (telephone or home visit) from a health care provider within 10 days of hospital discharge. Organisation and delivery of postnatal contact varied between birth facilities – in some cases, it was conducted by midwives from the birth facility directly, in other cases responsibility (and sometimes funding) for this service was transferred to community child health services. Variations in UPNCS delivery between birth facilities has been described elsewhere [32].

The UPNCS was funded in three stages. In June 2008, nine public birthing facilities were funded. In June 2009, a further 14 public birthing facilities were funded, and in June 2010 the remaining 18 public birthing facilities in Queensland were funded. Facilities were expected to have implemented the UPNCS within six months of receiving funding. The focus of this study is the impact of the third component of the initiative - provision of at least one postnatal contact to every woman – on women’s experience of postnatal contact, parenting confidence, and perceived sufficiency and quality of postnatal care.

### Design

The study comprised an observational cohort study with post-exposure assessment of outcomes.

### Measures

#### UPNCS funding

At the time of participants’ births, 23 public facilities had received UPNCS funding and were expected to have a fully implemented UPNCS. Women birthing in these facilities formed the ‘UPNCS-funded’ condition. Women birthing in the remaining 18 public facilities that had not yet received funding for UPNCS formed the comparison condition (‘Not UPNCS-funded’). A dichotomous variable representing UPNCS funding condition (1 = *UPNCS-funded*, 0 = *Not UPNCS-funded*) was generated for each woman based on self-reported birth facility.

#### Postnatal contact

Women were asked if they ‘were phoned by a midwife or nurse’ and/or ‘were visited at home by a midwife or nurse’ within 10 days of being at home with their baby. Receipt of a telephone call and/or a visit at home within 10 days was coded as *postnatal contact*, consistent with UPNCS definitions [17].

#### Number of postnatal telephone calls and home visits received

Women were asked how many times they had been ‘phoned by a health care provider’ and ‘visited at home by a health care provider’ since being at home or having their baby at home. The sum of reported number of home visits and telephone calls was calculated.

#### Parenting confidence

Women were asked how confident they felt looking after their new baby when he or she was brought home, with five response options. A top score analysis approach was adopted [33] such that ‘extremely confident’ was coded as *confident* and all other responses (from ‘fairly confident’, to ‘not at all confident’) were coded as *not confident*.

#### Perceived sufficiency of postnatal contact

Women were asked to rate the amount of postnatal contact they had with care providers as ‘too much’, ‘too little’ or ‘about right’. ‘Too little’ was coded as *insufficient amount* and ‘too much’ and ‘about right’ were coded as *sufficient* postnatal contact.

#### Perceived quality of postnatal care

Women were asked how well they felt they were looked after by care provider(s) after having their baby, on a five-point rating scale with anchors of ‘very badly’ and ‘very well’. Such evaluative assessments of care have been shown to have stronger associations with other indicators of care effectiveness, such as intentions to follow care provider advice or return for further care, than ‘satisfaction’ ratings [34]. Again, consistent with previous research [7,35,36], a top score analysis approach was adopted such that ‘*very well’* was coded as *high quality* postnatal care and all other responses coded as *not high quality*.

#### Other variables

Maternal age, parity, identification as Aboriginal and/or Torres Strait Islander, use of English at home, highest level of education, and area of residence were either recorded directly by women or derived from their responses. Distance travelled to birth was calculated as the distance from the suburb of maternal residence to the suburb of the birthing facility using geocoding software. Distance was categorised into *<10 km, 10-50 km, 50+ km* based on anecdotal reports from maternal and child health services in Queensland that postnatal health care providers will typically travel no further than 10-50 km to provide a home visit. Women were asked how many hours or days they stayed in hospital following birth (*length of hospital stay)*. Data were categorised into four groups; *<24 hours*, *1*–*2 nights*, *3*–*4 nights*, *5+ nights. 1*–*2 nights* was used the referent category as it is the modal length of postnatal stay in Queensland [37].

### Data analysis

Four multivariate binomial logistic regression analyses were conducted to assess the impact of UPNCS funding condition on the dependent variables: postnatal contact, parenting confidence, perceived sufficiency of postnatal contact and perceived quality of postnatal care, adjusting for potentially confounding demographic variables. Second, three multivariate binomial logical regression analyses were conducted to assess factors associated with parenting confidence, perceived sufficiency, and perceived quality of postnatal care. Only variables with a significant univariate association (p < 0.05) with the relevant dependent variable were included in each multivariate model and variables were entered simultaneously to adjust for all other significant variables. A p value of <0.05 was used for all analyses.

### Ethical approval

Ethical approval for the *Having a Baby in Queensland Survey, 2010* and subsequent analyses was obtained from The University of Queensland *Behavioural & Social Sciences Ethical Review Committee* (Clearance #2010000613). The UPNCS was implemented by Queensland Health as a service provision improvement activity, independent of this data collection.

## Results

### Participant flow

A total of 21,013 women gave birth during the sampling period. After excluding women due to experience of neonatal death (n = 67) or other reasons (e.g., incomplete mailing address; n = 39), 20,907 surveys were distributed. Of these, 543 surveys were returned to sender, resulting in delivery of survey packages to an estimated 20,364 women. Of the 7,194 (35.33%) women who returned a survey with usable data, 3,091 were excluded from this analysis because they did not birth in a public facility, leaving a sample of 4,103 women. Women were further excluded if they had data missing for parity (n = 18), Aboriginal and/or Torres Strait Islander identification (n = 27), use of English at home (n = 122), highest level of education (n = 24), area of residence (n = 60), distance travelled to birth (n = 22), length of stay in hospital (n = 19), postnatal contact (n = 10), parenting confidence (n = 45), perceived sufficiency of postnatal contact (n = 19), or perceived quality of postnatal care (n = 13). Missing values for maternal age, total telephone calls received and total home visits received exceeded five per cent (n = 215, n = 369, n = 279, respectively), so a subcategory of ‘missing’ was created for these variables and included in all analyses. The final sample for analysis included 3,724 women who had a live birth in a public hospital or birth centre in Queensland during the sampling period.

### Participant characteristics

Participants were aged between 15 and 48 years at the time of birth (*M* = 29.21 years, *SD* = 5.56 years). Demographic characteristics of the sample within each condition of UPNCS implementation and for the sample as a whole are shown in Table 1.

**Table 1:** **Demographic characteristics of the sample by UPNCS implementation**

	**UPNCS implementation**			**Total sample (N = 3,724)**
	**Not UPNCS-funded (N = 1,647)**	**UPNCS-funded (N = 2,077)**	**χ** ^**2**^ **(df), p**	
	**N (%)**	**N (%)**		**N (%)**
Maternal age (years)				
<20	50 (3.0%)	76 (3.7%)	17.951 (6), 0.006	126 (3.4%)
20 – 24	237 (14.4%)	364 (17.5%)		601 (16.1%)
25 – 29	496 (30.1%)	659 (31.7%)		1155 (31.0%)
30 – 34	478 (29.0%)	525 (25.3%)		1003 (26.9%)
35 – 39	262 (15.9%)	277 (13.3%)		539 (14.5%)
40 +	51 (3.1%)	65 (3.1%)		116 (3.1%)
Missing	73 (4.4%)	111 (5.3%)		184 (4.9%)
Parity				
Primiparous	745 (45.2%)	926 (44.6%)	0.157 (1), 0.692	1671 (44.9%)
Multiparous	902 (54.8%)	1151 (55.4%)		2053 (55.1%)
Aboriginal and/or Torres Strait Islander identification				
No	1615 (98.1%)	2009 (96.7%)	6.228 (1), 0.013	3624 (97.3%)
Yes	32 (1.9%)	68 (3.3%)		100 (2.7%)
English spoken at home				
No	14 (0.9%)	4 (0.2%)	8.254 (1), 0.004	18 (0.5%)
Yes	1633 (99.1%)	2073 (99.8%)		3706 (99.5%)
Highest level of education				
No formal qualifications	30 (1.8%)	35 (1.7%)	37.698 (4), 0.000	65 (1.7%)
Year 10 or equivalent	189 (11.5%)	279 (13.4%)		468 (12.6%)
Year 12 or equivalent	388 (23.6%)	480 (23.1%)		868 (23.3%)
Trade/diploma	500 (30.4%)	773 (37.2%)		1273 (34.2%)
University	540 (32.8%)	510 (24.6%)		1050 (28.2%)
Area of residence				
Major city	1304 (79.2%)	849 (40.9%)	579.910 (3), 0.000	2153 (57.8%)
Inner regional	237 (14.4%)	613 (29.5%)		850 (22.8%)
Outer regional	89 (5.4%)	520 (25.0%)		609 (16.4%)
Remote	17 (1.0%)	95 (4.6%)		112 (3.0%)
Distance travelled to birth				
<10 km	597 (36.2%)	944 (45.5%)	103.230 (2), 0.000	1541 (41.4%)
10 – 50 km	961 (58.3%)	891 (42.9%)		1852 (49.7%)
50+ km	89 (5.4%)	242 (11.7%)		331 (8.9%)

### Equivalence of groups

Women in the Not UPNCS-funded condition were older and less likely to identify as Aboriginal and/or Torres Strait Islander and to speak English at home than women in the UPNCS condition, although the actual differences were small (see Table 1). Women in the Not UPNCS-funded condition were more likely to have a University degree and to live in a major city, and less likely to have travelled <10 km or 50+ km than women in the UPNCS condition (see Table 1).

### Impact of UPNCS implementation

Women in the UPNCS-funded condition had almost twice the odds of receiving postnatal contact as women who birthed in facilities that were Not UPNCS-funded, after adjustment for other differences between conditions (see Table 2). UPNCS funding condition was not associated with parenting confidence, perceived sufficiency of postnatal contact, or perceived quality of postnatal care.

**Table 2:** **Associations between UPNCS implementation and outcomes of interest (N = 3,724)**

		**Unadjusted**		**Adjusted** ^**1**^	
	**%**	**Odds ratio**	**95% CI**	**Odds ratio**	**95% CI**
Received telephone call/home visit within 10 days					
Not UPNCS-funded	84.6	1		1	
UPNCS-funded	87.3	1.26*	1.04-1.52	1.80***	1.50-2.23
Confident in parenting ability					
Not UPNCS-funded	32.1	1		1	
UPNCS-funded	34.0	1.09	0.95-1.26	1.04	0.89-1.21
Perceived sufficient amount of postnatal contact					
Not UPNCS-funded	84.8	1		1	
UPNCS-funded	84.6	0.99	0.83-1.18	0.90	0.74-1.09
Perceived postnatal care to be high quality					
Not UPNCS-funded	47.2	1		1	
UPNCS-funded	49.0	1.07	0.94-1.22	1.00	0.87-1.16

*p<0.05. ***p < 0.001.

^1^Adjusted for maternal age, Aboriginal and/or Torres Strait Islander identification, English spoken at home, highest level of education, area of residence, and distance travelled to birth.

### Factors associated with parenting confidence

Women who received 3, 5 or 7 home visits had lower odds of parenting confidence than women who received none, after adjustment for all other significant factors (see Table 3). Amount of telephone contact (relative to having received no contact) was not associated with parenting confidence.

**Table 3:** **Factors associated with parenting confidence (N = 3,724)**

			**Unadjusted**		**Adjusted** ^**1**^	
	**N**	**% confident**	**Odds ratio**	**95% CI**	**Odds ratio**	**95% CI**
Telephone calls received						
0	900	34.3	1		1	
1	944	35.7	1.06	0.88-1.29	1.04	0.84-1.28
2	687	34.2	0.99	0.81-1.23	0.97	0.77-1.23
3	359	29.5	0.80	0.62-1.05	1.00	0.74-1.35
4	158	31.0	0.86	0.60-1.24	1.32	0.87-1.99
5	120	32.5	0.92	0.61-1.38	1.28	0.80-2.03
6 or more	187	23.0	0.57**	0.40-0.83	0.73	0.47-1.13
Missing	369	31.7	0.89	0.69-1.15	0.94	0.69-1.30
Home visits received						
0	1062	36.7	1		1	
1	659	35.7	0.96	0.78-1.17	0.87	0.70-1.09
2	712	32.4	0.83	0.68-1.01	0.84	0.67-1.05
3	460	28.0	0.67**	0.53-0.85	0.71*	0.54-0.94
4	161	26.7	0.63*	0.43-0.91	0.69	0.46-1.06
5	114	23.7	0.54**	0.34-0.84	0.54*	0.33-0.90
6	101	29.7	0.73	0.47-1.14	0.99	0.60-1.65
7 or more	176	24.4	0.56**	0.39-0.80	0.63*	0.40-0.98
Missing	279	38.4	1.07	0.82-1.41	1.06	0.76-1.46
Length of hospital stay						
1-2 nights	1633	39.1	1		1	
<24 hours	306	47.1	1.39**	1.09-1.77	1.38*	1.05-1.81
3-4 nights	1293	26.6	0.57***	0.48-0.66	0.72***	0.61-0.86
5+ nights	492	22.2	0.44***	0.35-0.56	0.59***	0.46-0.76
Parity						
Primiparous	1671	14.2	1		1	
Multiparous	2053	48.6	5.69***	4.83-6.69	5.04***	4.26-5.96
Aboriginal and/or Torres Strait Islander identification						
No	3624	32.8	1		1	
Yes	100	45.0	1.67*	1.12-2.50	1.50	0.96-2.34
Highest level of education						
No formal qualifications	65	43.1	1		1	
Year 10/equivalent	468	42.5	0.98	0.58-1.65	1.07	0.61-1.89
Year 12/equivalent	868	35.6	0.73	0.44-1.22	0.87	0.50-1.51
Trade/diploma	1273	34.1	0.68	0.41-1.13	0.87	0.50-1.50
University	1050	25.2	0.45**	0.27-0.74	0.54*	0.31-0.94

*p < 0.05, **p < 0.01, ***p < 0.001.

^1^The model as a whole accounted for between 15.3% (Cox and Snell R square) and 21.3% (Nagelkerke R squared) of the total variance in parenting confidence, and correctly classified 69.5% of cases.

Compared with women who stayed in hospital 1–2 nights after their birth, women who stayed less than 24 hours had higher odds of parenting confidence, and those who stayed 3 nights or more had lower odds of parenting confidence, after adjustment for other significant factors. Multiparous women had more than 5 times the odds of parenting confidence than primiparous women (see Table 3). Women who had completed university education had approximately half the odds of parenting confidence as women with no formal qualifications (see Table 3). Maternal age, use of English at home, area of residence, and distance travelled to birth were not associated with the odds of parenting confidence (data not shown).

### Factors associated with perceived sufficiency of postnatal contact

Number of postnatal telephone calls and postnatal home visits were significantly and independently associated with perceived sufficiency of postnatal contact (see Table 4). Women who received one telephone call had 1.46 times the odds of perceiving the amount of postnatal contact as sufficient than women who did not (95% CI:1.16-1.84), and women who received one home visit had 1.64 times the odds of perceived sufficiency than women who did not (95% CI: 1.26-2.14). The odds of perceived sufficient postnatal contact increased with the number of telephone calls until five were received, and generally increased with the number of home visits received. Length of hospital stay was not associated with perceived sufficiency of contact after adjustment for other significant variables (data not shown).

**Table 4:** **Factors associated with perceived sufficiency of postnatal contact (N = 3,724)**

			**Unadjusted**		**Adjusted** ^**1**^	
	**N**	**% satisfied**	**Odds ratio**	**95% CI**	**Odds ratio**	**95% CI**
Telephone calls received						
0	900	74.8	1		1	
1	944	81.6	1.49***	1.19-1.87	1.46**	1.16-1.84
2	687	87.8	2.42***	1.84-3.18	1.92***	1.44-2.55
3	359	93.6	4.93***	3.15-7.72	3.53***	2.23-5.58
4	158	94.3	5.58***	2.80-11.13	3.70***	1.83-7.48
5	120	96.7	9.78***	3.57-26.80	5.84**	2.09-16.34
6 or more	187	95.2	6.67***	3.36-13.25	2.98**	1.42-6.28
Missing	369	88.9	2.70***	1.89-3.86	1.59*	1.07-2.38
Home visits received						
0	1062	74.9	1		1	
1	659	84.1	1.77***	1.38-2.28	1.64***	1.26-2.14
2	712	85.5	1.99***	1.55-2.55	1.70***	1.30-2.23
3	460	92.2	3.96***	2.74-5.71	2.84***	1.93-4.19
4	161	93.2	4.58***	2.45-8.58	2.88**	1.51-5.52
5	114	93.0	4.45***	2.14-9.25	2.43*	1.14-5.20
6	101	95.0	6.45***	2.60-16.02	3.97**	1.55-10.21
7 or more	176	96.6	9.52***	4.17-21.73	5.48***	2.28-13.18
Missing	279	89.2	2.79***	1.86-4.17	2.22***	1.42-3.47
Length of hospital stay						
1-2 nights	1633	85.2	1			
<24 hours	306	89.5	1.49*	1.01-2.20	1.05	0.70-1.58
3-4 nights	1293	83.8	0.90	0.73-1.10	0.89	0.72-1.09
5+ nights	492	82.3	0.81	0.62-1.06	0.82	0.62-1.08
Highest level of education						
No formal qualifications	65	70.8	1		1	
Year 10/equivalent	468	82.5	1.94*	1.08-3.49	2.09*	1.12-3.91
Year 12/equivalent	868	83.4	2.08*	1.18-3.65	2.24**	1.22-4.11
Trade/diploma	1273	85.2	2.38**	1.37-4.16	2.51**	1.38-4.56
University	1050	86.9	2.73***	1.55-4.80	2.78**	1.52-5.10
Area of residence						
Major city	2153	83.7	1		1	
Inner regional	850	84.8	1.09	0.87-1.35	1.28*	1.02-1.62
Outer regional	609	88.0	1.43*	1.09-1.87	1.55**	1.13-2.12
Remote	112	83.0	0.95	0.57-1.58	1.36	0.78-2.38
Distance travelled to birth						
<10 km	1541	85.8	1		1	
10 – 50 km	1852	84.4	0.90	0.74-1.09	1.02	0.84-1.25
50+ km	331	80.7	0.69*	0.51-0.94	0.77	0.54-1.09

*p < 0.05, **p < 0.01, ***p < 0.001.

^1^The model as a whole accounted for between 6.4% (Cox and Snell R square) and 11.1% (Nagelkerke R squared) of the total variance in satisfaction with amount of postnatal contact, and correctly classified 84.8% of cases.

Odds of perceiving that postnatal contact was sufficient increased for women with the highest level of education relative to women with no formal qualifications, and women who lived in regional areas had higher odds of being satisfied than women who lived in a major city (see Table 4). Distance travelled to birth was not significantly associated with perceived sufficiency of contact after adjustment for other significant variables, nor were maternal age, use of English at home, identification as Aboriginal and/or Torres Strait Islander or parity (data not shown).

### Factors associated with perceived quality of postnatal care

Women who received one telephone call had 1.26 times the odds of perceiving their postnatal care as high quality than women who did not (95% CI: 1.04-1.52), and the odds increased further for women receiving two and three calls, but did not increase meaningfully with additional calls (see Table 5). Only women who received 6 or more home visits had significantly higher odds of perceived high quality postnatal care than women who received none. Women who stayed in hospital 1–2 nights after birth had significantly higher odds of perceiving postnatal care as high quality than women who stayed <24 hours.

**Table 5:** **Factors associated with perceived quality of postnatal care (N = 3,724)**

			**Unadjusted**		**Adjusted** ^**1**^	
	**N**	**% high quality**	**Odds ratio**	**95% CI**	**Odds ratio**	**95% CI**
Telephone calls received						
0	900	38.3	1		1	
1	944	43.6	1.25*	1.03-1.50	1.26*	1.04-1.52
2	687	49.6	1.59***	1.30-1.94	1.54***	1.25-1.90
3	359	58.5	2.27***	1.77-2.91	2.17****	1.67-2.81
4	158	57.6	2.19***	1.55-3.08	2.07***	1.45-2.97
5	120	58.3	2.25***	1.53-3.32	1.97**	1.30-2.97
6 or more	187	67.9	3.41***	2.44-4.76	2.30***	1.57-3.37
Missing	369	54.2	1.90***	1.49-2.43	1.66***	1.26-2.19
Home visits received						
0	1062	43.8	1		1	
1	659	42.6	0.95	0.78-1.16	0.86	0.70-1.06
2	712	44.1	1.01	0.84-1.23	0.85	0.69-1.04
3	460	53.7	1.49***	1.20-1.86	1.14	0.89-1.45
4	161	54.7	1.55*	1.11-2.16	1.10	0.77-1.57
5	114	55.3	1.59*	1.08-2.34	1.09	0.72-1.65
6	101	71.3	3.19***	2.04-4.99	2.16**	1.34-3.48
7 or more	176	68.2	2.75***	1.96-3.86	1.71**	1.16-2.52
Missing	279	52.3	1.41*	1.08-1.84	1.10	0.82-1.48
Length of hospital stay						
<24 hours	1633	48.5	1		1	
1-2 nights	306	61.4	1.69***	1.32-2.17	1.33*	1.02-1.73
3-4 nights	1293	46.2	0.91	0.79-1.06	0.95	0.82-1.11
5+ nights	492	44.3	0.85	0.69-1.04	0.85	0.68-1.05
Maternal age (years)						
<20	126	38.1	1		1	
20 – 24	601	42.6	1.21	0.81-1.79	1.20	0.79-1.80
25 – 29	1155	47.2	1.45	1.00-2.12	1.42	0.95-2.10
30 – 34	1003	51.6	1.74**	1.19-2.54	1.66*	1.11-2.47
35 – 39	539	52.9	1.82**	1.23-2.71	1.68*	1.10-2.56
40 +	116	49.1	1.57	0.94-2.62	1.37	0.80-2.35
Missing	184	47.3	1.46	0.92-2.31	1.35	0.83-2.18
Parity						
Primiparous	1671	44.3	1		1	
Multiparous	2053	51.4	1.33***	1.17-1.52	1.30***	1.12-1.51
Area of residence						
Major city	2153	46.5	1		1	
Inner regional	850	46.4	0.99	0.85-1.16	1.06	0.89-1.25
Outer regional	609	57.6	1.56***	1.30-1.87	1.59***	1.27-1.91
Remote	112	43.8	0.89	0.61-1.31	1.11	0.73-1.68
Distance travelled to birth						
<10 km	1541	48.9	1		1	
10 – 50 km	1852	48.6	0.99	0.86-1.13	1.02	0.89-1.18
50+ km	331	42.9	0.78*	0.62-1.00	0.71*	0.55-0.93

*p < 0.05, **p < 0.01, ***p < 0.001.

^1^The model as a whole accounted for between 5.6% (Cox and Snell R square) and 7.4% (Nagelkerke R squared) of the total variance in perceived quality of postnatal care, and correctly classified 59.2% of cases.

Multiparous women had significantly higher odds of perceiving their postnatal care as high quality than primiparous women and women who lived in outer regional areas had 1.59 times the odds of high quality care, after adjustment for other significant factors (95% CI: 1.27-1.91; see Table 5). Women who travelled more than 50 km to birth had significantly lower odds of perceived high quality care than women who travelled less than 10 km. Use of English at home, identification as Aboriginal and/or Torres Strait Islander, and highest level of education were not associated with perceived quality of postnatal care (data not shown).

## Discussion

The purpose of this study was to evaluate the impact of provision of funding for a universal postnatal contact service on women’s receipt of at least one contact in the immediate post-discharge period, and on parenting confidence, perceived sufficiency of postnatal contact, and perceived quality of postnatal care. Using data from a large population survey of postnatal women, we also examined the unique contribution of other factors to these outcomes, including the effects of small increments and modal differences (i.e., home visit vs. telephone call) in postnatal contact.

### Impact of UPNCS implementation

Women who birthed in a facility that had received UPNCS funding had almost twice the odds of receiving a telephone call and/or home visit within 10 days of being at home. This suggests that the provision of funding for UPNCS in Queensland was somewhat effective in advancing the proximal objective for all women to receive at least one postnatal contact. However, approximately 13% of women who birthed in facilities with UPNCS funding were not contacted and funding had no effect on women’s parenting confidence, their perceived sufficiency of postnatal care, or the perceived quality of their postnatal care.

We were unable to determine how variations in the timing of contact within the first 10 days of discharge may have impacted our outcome variables of interest, although findings from other studies suggest that the timing of contact is important. Goulet, D’Amour and Pineault found that the earlier women received a telephone call or home visit after discharge, the more likely they were to rate the service as useful [28]. The impact of timing may also interact with the type of contact provided. In other studies, women receiving a home visit within 48 hours of discharge had significantly higher appreciation [24,25] and satisfaction [25] than women receiving a clinic visit. However, when a home visit was compared to a clinic visit at 3–4 days post-discharge, this difference was not apparent [26]. Therefore, home visits may result in higher satisfaction than clinic visits in the immediate days following hospital discharge, but the type of contact may have less impact on women’s satisfaction after 3 days of being at home after birth. In this study, our cumulative measure of amount of postnatal contact in the 10 days following hospital discharge (i.e. any contact within the defined timing criterion of the UPNC initiative being evaluated) meant we were unable to separate the effects of earlier and later contact within that period. Further research is needed to determine the effect of variations in timing of contact, and the relative effects of telephone versus in-home support when timing of contact is equivalent.

### Amount of postnatal contact

We found no association between amount of telephone contact and parenting confidence, and that women who received a higher number of home visits had lower odds of parenting confidence than women who received none. Women who received a higher number of home visits may have been experiencing greater difficulties in the postnatal period, influencing both their parenting confidence and the number of home visits they received. Other research suggests that more frequent home visits (weekly from 10–14 days to eight weeks postpartum) have no impact on parenting confidence compared to one home visit within 10–14 days postpartum in low risk first time mothers [37].

Our results suggest a positive dose–response association between amount of postnatal contact and both perceived sufficiency of postnatal contact and perceived quality of postnatal care. Therefore, while UPNCS had no effect on these outcomes, strategies to increase the minimum amount of telephone contact provided for postpartum women may increase perceived sufficiency and quality of care.

The positive association between number of postnatal home visits and perceived quality of postnatal care was only evident when six or more home visits were received. This is consistent with findings of Christie and Bunting [38] who found that weekly home visits over six weeks significantly increased satisfaction compared to one home visit. Several home visits may be needed before noticeable effects on women’s perceptions of the quality of their postnatal care are realised, possibly due to increased opportunity for the postnatal care provider to develop a meaningful, supportive relationship that may not be possible with less contact. Home visits may be conducted by midwifery or child health service professionals, by individuals either previously known or unknown to women, and (for repeated visits) by the same or different care providers each time. Midwives and Child Health Nurses have different skill sets and relative emphasis on care of the mother and care of the baby. Future research should examine whether type and continuity of the postnatal carer providing home visiting moderates the association between home visits and perceived quality of postnatal care.

### Type of postnatal contact

Receiving one home visit resulted in only slightly higher odds of perceived sufficiency of contact than having one telephone call and, unlike telephone contact, had no effect on the perceived quality of postnatal care. Effects of different types of postnatal contact on perceived quality of postnatal care seem to interact with the amount received, possibly due to the different mechanisms by which telephone vs. home visiting can influence perceptions of quality of care. The quality of the interaction with the care provider, the content of discussions or education during contact, and the perceived benefits of different types of contact relative to their convenience may influence the impact of postnatal contact on perceived quality of care differently for different types of contact. Further research is needed to determine how telephone and home visiting contacts might differ in length, content, and women’s perceptions of the extent to which different modes of contact can meet their postnatal care needs.

### Length of hospital stay

Non-typical lengths of hospital stay – both shorter (<24 hours) and longer (3 nights or more) – were associated with decreased odds of women feeling confident taking care of their new baby at home. Longer hospital stays could indicate complications during birth or poor maternal or infant health, which could affect confidence and explain this finding. This finding could also point to a mediator effect, whereby mode of birth accounts for the association between hospital stay and confidence; women who had a caesarean section are likely to have had longer hospital stays and may also feel less confident about looking after their new baby than women who birthed vaginally (e.g., due to reduced mobility in the postpartum or other factors). Further assessment of the reasons for longer hospital stay in explaining associations with parenting confidence is needed, as is research to explore how postnatal services might mitigate the risks of longer hospital stay for parenting confidence.

### Limitations

This study was conducted as an opportunistic natural experiment to evaluate the impact of state funding to maternity health services for the provision of universal postnatal contact. While this was most useful for determining the likely impact of similar widespread funding initiatives to establish or extend universal postnatal care programs, it is also possible that facilities who had not received UPNCS funding at the time of data collection were already engaged in comparable postnatal care delivery. Thus, it is possible that these findings underestimate the effect of initiating universal postnatal contact services in settings where none are provided. Our findings regarding the predictors of parenting confidence and perceived sufficiency and quality of care are therefore helpful in allowing us to infer possible effects of introducing specific postnatal care recommendations where none is provided.

This study relied on retrospective recall more than four months after birth. Such data could be prone to recall bias, and reported confidence and evaluations of care may be affected by parenting experiences following the immediate postnatal contact period. Further, our measure of parenting confidence asked women to report on their experience ‘when first at home’ with their new baby, so it is possible that some women responded in reference to a period before post-discharge care was provided. Based on our pilot testing of the survey items, this is unlikely given the placement of the survey item alongside those asking women to report on their experience in the entire period since their birth. The sensitivity and validity of the one-item measure used to assess parenting confidence here is also unknown. We were motivated to use single-item measures by the need for brevity and minimised participant burden in a large survey intending to assess a wide range of experiences and outcomes, and encouraged by the success of short heath measures in other fields [39-43]. Nevertheless, our findings about the impact of UPNCS implementation and other features of postpartum care delivery on parenting confidence are speculative and attempted replication in future research is desirable.

There may have been other systematic differences between women birthing in UPNCS facilities and non-UPNCS facilities that we were unable to account for here. Studies that examine women’s experiences of postnatal care and parenting closer to the timing of the experience, that prospectively assess women’s experiences of care and associated outcomes, and that allow for randomisation to different protocols for postnatal care provision after discharge, may overcome these issues, and are a desirable extension of the current study.

### Future research and recommendations

Future research should seek to understand how more specific features of postnatal contact influence the outcomes studied here. For example, how does the length and content of the contact influence women’s confidence and evaluations of care? What is it about postnatal contact that women value? How does continuity of care provider influence the effectiveness of postnatal care delivery? These factors have not been examined in the existing universal postnatal care program literature, but are likely to vary substantially from program to program. Redressing these gaps in knowledge would provide further insight into the most effective components of postnatal care provision.

## Conclusions

A number of preliminary recommendations can be made for enhancing women’s satisfaction with postnatal care based on the findings reported here. First, resources should be directed toward increasing the minimum number of postnatal contacts women receive. Second, it is tentatively recommended that women receive at least three telephone or home visit contacts postnatally to optimise perceived sufficiency of care provided. Third, telephone contact may offer the most cost-effective mode of postnatal care delivery for maximising the quality of postnatal care from women’s perspective. In circumstances where only one postnatal contact can be provided, a home visit is unlikely to result in substantially greater benefits than telephone contact.

Further service improvements are likely to be necessary to produce population-level improvements in parenting confidence. Our findings suggest that women having their first baby, those who have longer postpartum hospital stays, and those with higher levels of education are most at risk of low postpartum parenting confidence, irrespective of other factors including postnatal contact. We need to better understand the relative costs and benefits of postnatal care policies and services that are universal, or that selectively target these at-risk women, to inform population approaches for improving maternal and infant well-being. In the future, similar policy initiatives would be better served by preliminary evaluation in small-scale efficacy trials to better inform recommendations for cost-effective implementation of universal postnatal care services prior to significant investment.
